# Prognostic value of surgical treatment in elderly patients with ulcerative colitis‐associated colorectal cancer: A subanalysis of a nationwide Japanese multicenter study

**DOI:** 10.1002/ags3.12885

**Published:** 2024-11-12

**Authors:** Kinuko Nagayoshi, Yusuke Mizuuchi, Masafumi Nakamura, Koji Okabayashi, Motoi Uchino, Hiroki Ikeuchi, Tatsuki Noguchi, Soichiro Ishihara, Yoichi Ajioka, Kenichi Sugihara, from the Study Group for Inflammatory Bowel Disease Associated Intestinal Cancers by the Japanese Society for Cancer of the Colon and Rectum

**Affiliations:** ^1^ Department of Surgery and Oncology, Graduate School of Medical Sciences Kyushu University Fukuoka Japan; ^2^ Department of Surgery Keio University Tokyo Japan; ^3^ Department of Inflammatory Bowel Disease Surgery Hyogo Medical University Nishinomiya Japan; ^4^ Department of Surgical Oncology The University of Tokyo Tokyo Japan; ^5^ Division of Molecular and Diagnostic Pathology, Graduate School of Medical and Dental Sciences Niigata University Niigata Japan; ^6^ Department of Surgical Oncology Tokyo Medical and Dental University Tokyo Japan

**Keywords:** elderly patient, recurrence, surgery, ulcerative colitis‐associated colorectal cancer

## Abstract

**Aim:**

Our study aimed to examine the characteristics of elderly patients diagnosed with ulcerative colitis‐associated colorectal cancer (UAC) and evaluate the impact of surgical intervention on prognosis.

**Methods:**

A total of 1086 patients diagnosed with UAC between 1980 and 2020 were retrospectively enrolled. Data were collected through the Japanese Society for Cancer of the Colon and Rectum project. The patients were divided into two groups: 248 elderly patients in the E‐UAC group and 838 nonelderly patients in the NE‐UAC group. Patients aged >65 y at cancer diagnosis were considered elderly.

**Results:**

The recurrence rate did not differ between the two groups. Segmental resection was the only common independent risk factor for recurrence in both the E‐UAC and the NE‐UAC groups. The E‐UAC patients had significantly better 5‐y disease‐specific survival (DSS) than the NE‐UAC patients (94.7% vs 91.0%, *p* = 0.04). There were no differences in 5‐y recurrence‐free survival (RFS; 89.3% vs 86.6%, respectively, *p* = 0.24) or overall survival (OS; 88.8% vs 89.6%, *p* = 0.50). The E‐UAC patients who underwent segmental resection had poorer RFS than those who underwent total proctocolectomy, but there were no significant differences in DSS or OS.

**Conclusion:**

Despite the elevated risk of cancer recurrence observed in the UAC patients who underwent segmental resection in both the E‐UAC the NE‐UAC groups, our findings suggested that segmental resection may be a viable alternative to total proctocolectomy in terms of survival rate for the E‐UAC patients.

## Graphical abstract

Our study examined the characteristics of elderly patients diagnosed with ulcerative colitis‐associated colorectal cancer. We showed that factors associated with aggressive tumor characteristics played a significant role in recurrence risk in the nonelderly patients, but not in the elderly patients.

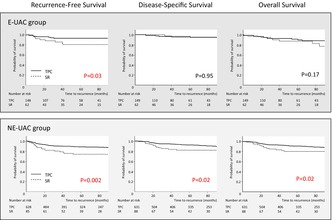

## 1 INTRODUCTION

Ulcerative colitis (UC) is a common inflammatory bowel disease (IBD) in young adults. The incidence of IBD is rising among children and older adults in developed countries, contributing to the reported increase in the number of elderly people affected.^1^ The prevalence of IBD—including UC—has increased significantly in Japan over the past two decades.^2^ Because Japan has a large aging population, the number of elderly patients with UC has also increased. Although the clinical characteristics and disease course of UC were assumed to be similar in elderly and nonelderly individuals, studies have suggested that elderly patients with UC have significant clinical differences in disease activity and surgery rates compared with younger patients.^3^, ^4^ Additionally, it is well known that patients with longer disease duration and extensive UC have an increased risk of developing colorectal cancer.^5^ However, few reports have described clinical differences between elderly patients with UC‐associated colorectal cancer (E‐UAC) and nonelderly patients with UC‐associated colorectal cancer (NE‐UAC). Furthermore, few studies have reported the impact of surgical treatments on postoperative cancer prognosis in E‐UAC. Given the growing number of elderly patients with UC, it is crucial to understand the prognostic impact of surgery on these individuals. This information can influence patient management by allowing clinicians to provide tailored treatment that considers the balance between invasiveness, cancer prognosis, and quality of life (QOL).

Our study aimed to evaluate the clinical characteristics of the E‐UAC population and examine the prognostic impact of surgical intervention. This retrospective study compared clinicopathological characteristics and surgical outcomes between E‐UAC and NE‐UAC populations using the large national database from the Japanese Society for Cancer of the Colon and Rectum (JSCCR).

## 2 METHODS

### 2.1 Patients and data collection

**FIGURE 1:**
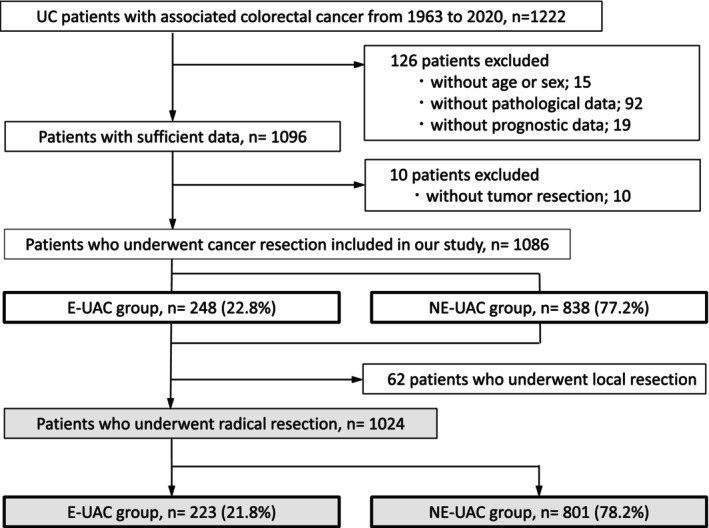
Flowchart describing patient selection. UC, ulcerative colitis; E‐UAC, elderly ulcerative colitis‐associated colorectal cancer; NE‐UAC, nonelderly ulcerative colitis‐associated colorectal cancer.

A total of 1222 patients diagnosed with UC‐associated colorectal cancer (UAC) between 1980 and 2020 were retrospectively identified and enrolled in this study. Patient data were collected from 43 institutions that participated in the JSCCR project.^6^ Of the enrolled patients, 126 had missing clinical data so could not be analyzed and 10 did not receive tumor resection. Finally, 1086 patients were included in the study (Figure 1). The 1086 patients were divided into two groups based on age: 248 patients aged over 65 y at cancer diagnosis were placed in the E‐UAC group and 838 patients aged under 65 y at diagnosis comprised the NE‐UAC group.

Patient characteristics collected for the analysis included the following: age at cancer diagnosis; sex; disease duration; presence of primary sclerosing cholangitis (PSC); initial symptoms; colitis classification; clinical course^7^; diagnostic method; indication for surgery; location of main tumor; clinical tumor (T), node (N), and metastasis (M) stage; surgical findings including procedure, outcomes, postoperative complications, and curability; histopathological findings in resected specimens including histology and pathological TNM stage; lymphatic and venous invasion^8^; node involvement; distant metastasis; use of adjuvant chemotherapy; postoperative complications; site of recurrence; and prognosis. Postoperative complications were evaluated using the Clavien–Dindo classification. Patients were followed up for a median of 53.1 mo (range: 1–535).

### 2.2 Statistical analysis

Univariate analyses were conducted to compare the two groups. All statistical analyses were performed using JMP Pro v 17.0.0 software (SAS Institute, Cary, NC, USA). Clinical and demographic characteristics were analyzed using the *χ* ^2^ test for categorical variables. Differences in continuous variables were compared using the Mann–Whitney *U* test. One‐way analysis of variance was used to compare the remaining continuous variables. Logistic regression analysis was used to determine independent risk factors for recurrence. In the multivariate analysis, all variables were included in a backward stepwise multiple logistic regression to identify significant risk factors for cancer recurrence. The final model included the surgical approach and adjuvant chemotherapy in the E‐UAC group, histological residual tumor, tumor differentiation, pathological N status, venous invasion, and lymphatic invasion in the NE‐UAC group. The fitness of the model was evaluated by a likelihood ratio test showing *p <* 0.001. Survival analysis was performed using the Kaplan–Meier method and results were compared using the log‐rank test. *p*‐values of <0.05 were considered statistically significant.

## 3 RESULTS

### 3.1 Comparison of characteristics between the E‐UAC and NE‐UAC groups

Table 1 shows a comparison of the clinical features between the E‐UAC and NE‐UAC groups. There were no significant differences between the groups in the proportion of males and females, disease duration, the presence of PSC, or UC classification. In the E‐UAC group, 20.3% were diagnosed with UC when they were over 65 y of age (elderly‐onset). While ~25% of patients in each group had initial symptoms at colorectal cancer diagnosis, more than 65% in each group were diagnosed incidentally during surveillance. More patients in the E‐UAC than the NE‐UAC group had a clinically chronic continuous disease course (25.0% vs 21.2%) and fewer patients had a relapse–remission course (61.3% vs 70.2%, *p* = 0.02). In the E‐UAC group, 26.0% of patients with elderly‐onset UAC had a clinically chronic, continuous disease course, which was consistent with the proportion observed in all patients in the E‐UAC group. Endoscopy was the most frequent modality for cancer diagnosis in both groups (95.1% and 93.5%, respectively). The distribution of left‐ and right‐sided tumors was similar in the two groups.

**TABLE 1:** Comparison of clinical characteristics between the E‐UAC and NE‐UAC groups.

Characteristic		E‐UAC group	NE‐UAC group	*p*‐value
		*N* = 248	*N* = 838	
Sex, *n* (%)	Male/female	160/88 (64.5%/35.5%)	513/325 (61.2%/38.8%)	0.35
Median disease duration, mo (range)		185 (0–684)	192 (0–590)	0.81
Age >65 at UC onset, *n* (%)		50 (20.3%)	‐	
Presence of PSC, *n* (%)		4 (1.6%)	23 (2.7%)	0.25
Cancer diagnosis, *n* (%)	After initial symptoms	60 (24.2%)	220 (26.3%)	0.38
	During surveillance	171 (69.2%)	546 (65.5%)	
UC classification, *n* (%)	Extended	184 (74.1%)	650 (77.6%)	0.23
	Lest‐sided	44 (17.7%)	150 (17.9%)	
	Proctitis	9 (3.6%)	19 (2.3%)	
	Unknown	11 (4.4%)	19 (2.2%)	
Clinical course, *n* (%)	Relapse‐remission	152 (61.3%)	588 (70.2%)	0.02*
	Chronic continuous	62 (25.0%)	178 (21.2%)	
	Other	34 (13.7%)	72 (8.6%)	
cStage (UICC), *n*	0/I/II/III/IV	76/80/45/20/3	219/251/116/105/18	0.10
Tumor location	Left side	177 (79.4%)	670 (83.7%)	0.33
	Right side	43 (19.3%)	121 (15.1%)	
	Unknown	3 (1.3%)	10 (1.2%)	
Surgical treatment, *n* (%)	Radical resection	223 (89.9%)	801 (96.6%)	0.002*
	Local resection	25 (10.1%)	37 (4.4%)	

Abbreviations: cStage, clinical stage; E‐UAC, elderly ulcerative colitis‐associated colorectal cancer; NE‐UAC, nonelderly ulcerative colitis‐associated colorectal cancer; PSC, primary sclerosing cholangitis; UC, ulcerative colitis; UICC, Union for International Cancer Control.

* Statistically significant (*p* < 0.05).

Of the 1086 patients, 62 (5.7%) had a local resection, that is, tumor resection without lymphadenectomy such as endoscopic resection, transanal resection, and appendectomy. Local resection was performed in 25 patients (10.1%) in the E‐UAC group and 37 patients (4.4%) in the NE‐UAC group (*p* = 0.002). Additional surgical resection was required by four (16.0%) and 13 patients (41.9%), respectively (*p* = 0.03). The 62 patients who underwent local resection included 15 stage 0 patients, 45 stage I patients, and two stage IV patients. Except for stage IV patients, all patients who underwent local resection had no tumor recurrence.

Of the 1086 patients, 1024 underwent radical resection for colorectal cancer, that is, en‐bloc resection of the entire anatomical compartment containing the tumor. Patients who received radical resection included 223 in the E‐UAC group and 801 in the NE‐UAC group (Figure 1). A comparison of the surgical procedures and short‐term outcomes between the E‐UAC and NE‐UAC groups is shown in Table 2. The surgical approaches used did not differ between the two groups. Although total proctocolectomy (TPC) is the standard treatment for UAC, segmental resection (SR) was performed as a substitute for TPC more often in the E‐UAC group than in the NE‐UAC group (30.4% vs 13.9%, *p <* 0.001). SR included procedures with lymphadenectomy other than TPC, such as subtotal colectomy, hemicolectomy, partial colectomy, and anterior resection. Among patients treated with SR, less extensive procedures such as hemicolectomy and anterior resection tended to be selected for the E‐UAC group compared with the NE‐UAC group (64.2% vs 50.5%, *p* = 0.07). Among patients treated with TPC, 63 (40.7%) in the E‐UAC group had permanent ileostomy without reconstruction compared with only 115 patients (16.7%) in the NE‐UAC group (*p <* 0.001). There was no significant difference in postoperative complications (all grades) between the two groups. R0 resection was achieved more frequently in the E‐UAC group than in the NE‐UAC group (98.6% vs 94.8%, *p* = 0.02). Adjuvant chemotherapy was administered less frequently in the E‐UAC group than in the NE‐UAC group (10.8% vs 24.4%, respectively, *p* < 0.001); similarly, among pathological stage II and III patients, it was less frequently administered in the E‐UAC than NE‐UAC group (39.4% vs 73.7%, respectively, *p* < 0.001).

**TABLE 2:** Comparison of surgical procedures and outcomes between patients in the E‐UAC and NE‐UAC groups who underwent radical resection.

Procedure		E‐UAC group	NE‐UAC group	*p* value
		*N* = 223	*N* = 801	
Approach, *n* (%)	Open surgery	121 (54.3%)	448 (55.9%)	0.43
	Minimally invasive surgery	98 (44.9%)	346 (43.2%)	
	Unknown	4 (1.8%)	7 (0.9%)	
Procedure, *n* (%)	Total proctocolectomy	155 (69.5%)	687 (85.8%)	<0.001*
	Segmental resection^a^	67 (30.4%)	111 (13.9%)	
	Other	1 (0.4%)	3 (0.4%)	
Details of segmental resection^a^	Subtotal colectomy	24 (10.8%)	55 (6.9%)	0.07
	Hemicolectomy or anterior resection	43 (19.3%)	56 (7.0%)	
Reconstruction after TPC, *n* (%)	IAA	60 (38.7%)	466 (67.8%)	<0.001*
	IACA	32 (20.7%)	106 (15.4%)	
	Permanent ileostomy	63 (40.7%)	115 (16.7%)	
R0 resection, *n* (%)		215 (98.6%)	751 (94.8%)	0.02*
Postoperative complication, *n* (%)	All grades	65 (29.4%)	264 (33.3%)	0.28
Adjuvant chemotherapy, *n* (%)		24 (10.8%)	192 (24.4%)	<0.001*

Abbreviations: E‐UAC, elderly ulcerative colitis‐associated colorectal cancer; IAA; ileal pouch‐anal anastomosis, IACA; ileal pouch‐anal canal anastomosis; NE‐UAC, nonelderly ulcerative colitis‐associated colorectal cancer; TPC, total proctocolectomy.

* Statistically significant (*p* < 0.05).

^a^ Including subtotal colectomy, hemicolectomy, partial colectomy, and anterior resection.

Pathological findings showed that the E‐UAC group had significantly fewer undifferentiated tumors compared with the NE‐UAC group (6.9% vs 19.4%, *p* < 0.001) (Table 3). Lymph node metastasis and lymphatic invasion were less frequent in the E‐UAC group compared with the NE‐UAC group, as shown in Table 3. The pathological findings observed in the elderly‐onset patients were similar to those observed in all patients in the E‐UAC group (undifferentiated tumors, 2.1%; lymphovascular invasion, 31.9%). There were no significant differences between the E‐UAC group and the NE‐UAC group in the pathological stage or the proportions of patients with positive resection margins or multiple lesions.

**TABLE 3:** Comparison of pathological findings between the E‐UAC and NE‐UAC groups.

Finding		E‐UAC group	NE‐UAC group	*p* value
		N = 223	N = 801	
Pathological type, *n* (%)	Differentiated	189 (87.1%)	564 (73.3%)	<0.001*
	Undifferentiated	15 (6.9%)	150 (19.4%)	
pT, *n*	T0–2/3–4/X	144/73/6	483/292/26	0.27
pN, *n*	N0–1/2–3/X	210/7/6	699/88/14	<0.001*
Presence of lymphatic invasion, *n* (%)		60 (27.4%)	284 (36.6%)	0.03*
Presence of venous invasion, *n* (%)		53 (24.2%)	248 (32.0%)	0.06
pStage, *n*	0/1/2/3/4/X	69/65/40/38/4/7	222/222/146/148/37/26	0.40
Resection margins, *n* (%)	Positive	3 (1.4%)	18 (2.3%)	0.60
Multiple lesions, *n* (%)		50 (23.2%)	215 (27.5%)	0.20

Abbreviations: E‐UAC, elderly ulcerative colitis‐associated colorectal cancer; N, node; NE‐UAC, nonelderly ulcerative colitis‐associated colorectal cancer; p, pathological; T, tumor.

* Statistically significant (*p* < 0.05).

### 3.2 Tumor recurrence and survival prognosis

To assess differences in cancer prognosis between the two groups, we conducted Cox proportional hazards regression analyses for tumor recurrence. Among 950 patients with stage 0–III disease, 119 (12.5%) experienced recurrence; this included 19 of 212 patients in the E‐UAC group versus 100 of 738 in the NE‐UAC group (9.0% vs 13.6%, *p* = 0.07). While liver metastasis was less frequent in the E‐UAC group than in the NE‐UAC group (0.5% vs 2.9%, *p* = 0.02), there were no significant differences between groups in the patterns of local, lymph node, lung, and peritoneal recurrence (Data S1). Multivariate analysis revealed that SR (hazard ratio [HR], 3.47; 95% confidence interval [CI], 1.16–10.33, *p* = 0.03) and administration of adjuvant chemotherapy (HR, 3.81; 95% CI, 1.07–13.54, *p* = 0.04) were independent risk factors for recurrence among patients in the E‐UAC group (Table 4). In the NE‐UAC group, however, SR (HR, 2.15; 95% CI, 1.1–4.14, *p* = 0.02), positive residual tumor (HR, 4.67; 95% CI, 1.11–19.60, *p* = 0.04), pN2–3 (HR, 3.86; 95% CI, 1.90–7.88, *p <* 0.001), venous invasion (HR, 1.89; 95% CI, 1.01–3.54, *p* = 0.05), and lymphatic invasion (HR, 2.26; 95% CI, 1.17–4.38, *p* = 0.02) were independent risk factors for recurrence (Table 4). SR was the only common risk factor for recurrence between the two groups.

**TABLE 4:** Risk factors for recurrence after surgery in the E‐UAC and NE‐UAC groups.

	E‐UAC group				NE‐UAC group			
	Univariate analysis		Multivariate analysis		Univariate analysis		Multivariate analysis	
	HR (95%CI)	*p*‐value	HR (95%CI)	*p*‐value	HR (95%CI)	*p*‐value	HR (95%CI)	*p*‐value
Male/female					1.51 (0.96–2.37)	0.07		
Open/MIS					1.61 (1.04–2.50)	0.03*		
SR/TPC	2.99 (1.20–7.44)	0.02*	3.47 (1.16–10.33)	0.03*	2.86 (1.71–4.80)	<0.001*	2.15 (1.11–4.14)	0.02*
Residual tumor positive/negative					13.62 (4.46–41.57)	<0.001*	4.67 (1.11–19.60)	0.04*
Undifferentiated/differentiated					4.51 (2.84–7.18)	<0.001*		
pT3–4/T0–2	5.85 (2.25–15.25)	<0.001*			5.41 (3.44–8.51)	<0.001*		
pN2–3/N0–1	51.00 (5.37–483.7)	<0.001*			13.70 (7.89–23.78)	<0.001*	3.86 (1.90–7.88)	< 0.001*
Venous invasion, (positive/negative)	4.17 (1.63–10.68)	0.003*			6.08 (3.87–9.57)	<0.001*	1.89 (1.01–3.54)	0.05*
Lymphatic invasion, (positive/negative)	4.07 (1.59–10.38)	0.003*			6.89 (4.29–11.07)	<0.001*	2.26 (1.17–4.38)	0.02*
Adjuvant chemotherapy, (yes/no)	3.32 (1.10–10.10)	0.03*	3.81 (1.07–13.54)	0.04*	5.11 (3.29–7.93)	<0.001*		

Abbreviations: E‐UAC, elderly ulcerative colitis‐associated colorectal cancer; MIS, minimally invasive surgery; N, node; NE‐UAC, nonelderly ulcerative colitis‐associated colorectal cancer; p, pathological; SR, segmental resection; T, tumor; TPC, total proctocolectomy.

* Statistically significant (*p* < 0.05).

**FIGURE 2:**
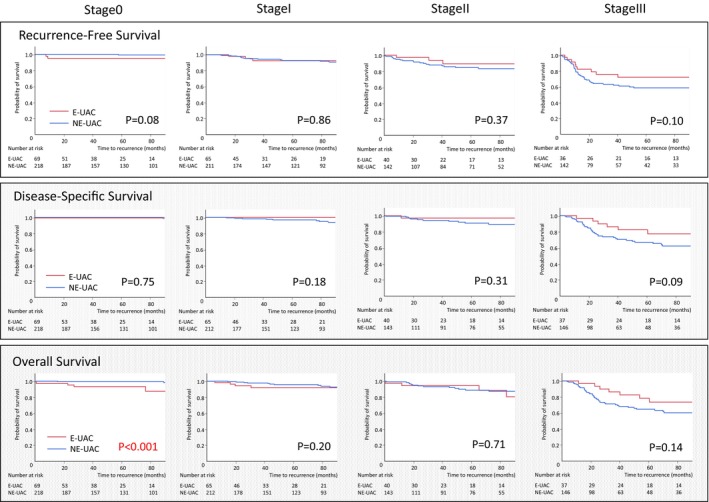
Comparison of survival between the E‐UAC and NE‐UAC groups according to cancer stage. E‐UAC, elderly ulcerative colitis‐associated colorectal cancer; NE‐UAC, nonelderly ulcerative colitis‐associated colorectal cancer.

Among 950 patients with stage 0–III disease, there were no significant differences between the E‐UAC and NE‐UAC groups in 5‐y recurrence‐free survival (RFS; 89.3% vs 86.6%, *p* = 0.24) or 5‐y overall survival (OS; 88.8% vs 89.6%, *p* = 0.50). However, patients in the E‐UAC group had significantly better 5‐y disease‐specific survival (DSS) compared with those in the NE‐UAC group (94.7% vs 91.0%, *p* = 0.04). Examining pathological stage, patients with stage 0 disease in the E‐UAC group had significantly poorer 5‐y OS than those in the NE‐UAC group (93.6% vs 99.5%, *p <* 0.001). There were no significant differences in 5‐y RFS (96.6% vs 99.2%, respectively, *p =* 0.08) or DSS (100.0% vs 97.0%, respectively, *p* = 0.75) (Figure 2). Patients in the E‐UAC group with stage III disease had a better prognosis according to 5‐y RFS, DSS, and OS compared with those in the NE‐UAC group, but the differences were not significant (RFS, 72.0% vs 58.4%, *p* = 0.10, DSS; 77.4% vs 66.8%, *p =* 0.09, OS; 78.4% vs 64.6%, *p* = 0.14) (Figure 2).

### 3.3 Subanalysis of survival prognosis by surgical procedure

**FIGURE 3:**
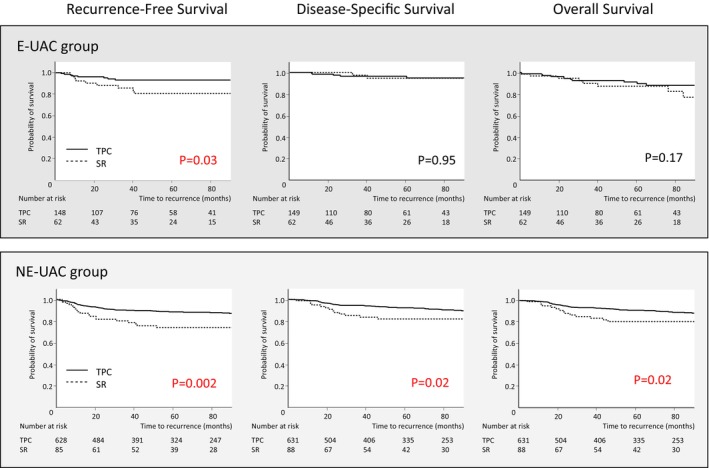
Subanalysis of survival prognosis by surgical procedure in the E‐UAC and NE‐UAC groups. E‐UAC, elderly ulcerative colitis‐associated colorectal cancer; NE‐UAC, nonelderly ulcerative colitis‐associated colorectal cancer; TPC, total proctocolectomy; SR, segmental resection.

We analyzed the association between surgical procedures and prognosis in each group, since it was the only common independent risk factor for recurrence. The analysis included 950 patients with stage 0–III disease. SR was performed more frequently than TPC for right‐sided cancer in both groups (E‐UAC group, 31.3% vs 14.1%, *p* = 0.01; NE‐UAC group, 29.7% vs 12.8%, *p* < 0.001). In both the E‐UAC and the NE‐UAC groups, patients who underwent SR had more pathological risk factors for recurrence, such as T3/4 tumor and lymphovascular involvement, resulting in more frequent tumor recurrence than those who underwent TPC (Data S1). There was no significant difference in the recurrence pattern between the patients who underwent SR and those who underwent TPC in the E‐UAC group. Meanwhile, patients in the NE‐UAC group who underwent SR had a significantly higher incidence of peritoneal metastasis compared with those who underwent TPC (10.0% vs 2.9%, *p* < 0.01, Data S1). The results also showed that patients who underwent SR had a significantly poorer prognosis compared with those who underwent TPC according to 5‐y RFS (89.2% vs 76.4%, *p <* 0.001) and OS (90.7% vs 83.0%, *p <* 0.001), but not DSS (92.7% vs 86.8%, *p* = 0.11). While patients in the E‐UAC group who underwent SR had poorer RFS than those who underwent TPC (80.6% vs 93.2%, *p =* 0.03), there were no significant differences in 5‐y DSS (94.6% vs 94.8%, *p* = 0.95) or OS (87.1% vs 89.5%, *p* = 0.17) (Figure 3). In the NE‐UAC group, patients who underwent SR had a significantly poorer prognosis compared with those who underwent TPC according to all 5‐y survival measures (RFS, 73.8% vs 88.4%, *p =* 0.002; DSS, 81.8% vs 92.2%, *p =* 0.02; OS, 80.3% vs 90.9%, *p =* 0.02) (Figure 3).

## 4 DISCUSSION

To our knowledge, this is the largest population‐based study to date comparing the characteristics and prognosis of elderly and nonelderly people with UAC. In the present study, we found several clinicopathologic differences, such as clinical course, surgical procedure, pathological differentiation of the tumor, N status, and lymphatic invasion between patients in the E‐UAC and NE‐UAC groups. Segmental resection was the only common independent risk factor for recurrence in the two groups. The E‐UAC patients who underwent segmental resection had poorer RFS than those who underwent total proctocolectomy, but there were no significant differences in DSS or OS. Our findings indicate that, despite the increased risk of cancer recurrence observed in the UAC patients who underwent segmental resection in both groups, segmental resection may be a viable alternative to total proctocolectomy, with comparable survival rates for E‐UAC patients.

Previous studies that examined differences in the characteristics of patients with UC reported that elderly individuals commonly have left‐sided UC at diagnosis and that they are less likely to have extensive disease and isolated proctitis than younger adults with UC.^9^, ^10^, ^11^ However, we found no such differences in the distribution of UC between the E‐UAC and NE‐UAC groups. The site of disease in elderly patients with UC tends to remain stable, with only a small proportion showing disease extension at follow‐up.^9^ Sugita et al found that patients with left‐sided colitis developed both their colitis and cancer about a decade later than those with extensive disease.^12^ Although elderly patients with UC generally have left‐sided colitis, our study showed that extensive colitis was more frequent than left‐sided colitis in patients in both the E‐UAC and NE‐UAC groups.

Regarding onset, previous studies suggested that disease activity may influence the development of colitis‐associated colorectal cancer, and older age at IBD onset may lead to the progressive development of colorectal cancer.^4^, ^13^ Continuous and extensive inflammation due to long‐standing disease is a risk factor for the development of colorectal cancer.^14^, ^15^ Patients with IBD‐associated colorectal cancer induced by inflammation have been characterized by an increased rate of poor histological features such as poor differentiation and mucinous or signet ring cell carcinoma.^16^ In our study, 20.3% of patients in the E‐UAC group had elderly‐onset disease. Therefore, our results are reflective of the majority of patients in the E‐UAC group, rather than those with elderly‐onset disease. In terms of disease course, more patients in the E‐UAC group had chronic continuous disease and fewer had a relapse–remission course compared with the NE‐UAC group. Furthermore, our pathological findings showed fewer poorly differentiated tumors and less lymphovascular invasion in the E‐UAC group, with similar findings in the elderly‐onset patients. Accordingly, we indicated that the clinicopathological characteristics of IBD‐associated colorectal cancer had less impact on tumor characteristics in most of the E‐UAC group than it did in the NE‐UAC group.

Previous studies have reported that most patients with IBD are diagnosed at an earlier cancer stage, which should have a better prognosis; nevertheless, all these patients, in particular for nodal disease, stage III, tend to have worse survival compared with sporadic cases of colorectal cancer.^17^, ^18^, ^19^ Patients with UAC also have similar characteristics; however, the majority are diagnosed at a relatively young age^12^ and the prognostic characteristics of the aged UAC population have not been fully evaluated. Our study found no significant difference in the recurrence rate by age, but cancer death was less frequent in the E‐UAC group than in the NE‐UAC group. Factors associated with aggressive histological subtype, lymphatic invasion, and venous invasion and N2‐3, played a significant role in the risk of recurrence in the NE‐UAC group, but not in the E‐UAC group, which showed better DFS and OS than the NE‐UAC group. This suggests that poor prognostic subtypes may be less prevalent among patients over 65 y of age and have less impact on their cancer prognosis.

While adjuvant chemotherapy has been established as standard treatment for advanced colorectal cancer, it is sometimes withheld from elderly patients after surgery when considering the optimal balance among their general status, the risk of recurrence, and disadvantage of chemotherapy.^19^ Patients with stage III disease in the E‐UAC group had better survival outcomes than those in the NE‐UAC group, despite being less likely to receive adjuvant chemotherapy. Meanwhile, the administration of adjuvant chemotherapy was identified as a significant risk factor for recurrence in the E‐UAC group. This finding may be attributed to the inherent limitations of the dataset, which consisted of a disproportionate number of stage 0 and I patients, representing nearly half of the E‐UAC group.

With regard to the surgical approach, we observed that patients in the NE‐UAC group who underwent SR instead of TPC had a higher recurrence rate and a poorer prognosis. In the E‐UAC group, patients who underwent SR had poorer RFS than those who underwent TPC, although there were no significant differences in DSS and OS. It is possible that the early censoring of patients due to a cause of death other than cancer may have had an impact on DSS and OS in the E‐UAC group. Additionally, the discrepancy in the recurrence pattern may be a contributing factor. In the NE‐UAC group, patients who underwent SR had a higher incidence of peritoneal metastasis compared with those who underwent TPC; however, this was not observed in the E‐UAC group. Patients with peritoneal metastases in the NE‐UAC group may have negatively affected the prognosis of patients in this group who underwent SR compared with those who underwent TPC, and compared with patients in the E‐UAC group. TPC is usually the standard procedure for radical resection of patients with UAC because it eliminates the possibility of tumor origin at another colorectal site. However, in elderly patients with IBD, factors such as disease severity, comorbidities, and QOL should be considered when selecting the appropriate surgical approach.^11^ In our study, ~30% of patients in the E‐UAC group were candidates for SR as an alternative to TPC to ensure safety and maintain their postoperative QOL, considering their general condition. A population‐based study of IBD in patients who underwent surgery for colorectal cancer found no significant difference in OS between patients who underwent SR and those who underwent TPC, suggesting that in selected patients with IBD‐associated colorectal cancer, SR may not compromise survival outcomes.^20^ Previous studies found that SR did not increase the risk of metachronous cancer or distant recurrence in elderly patients and indicated that it may be a viable option for colorectal cancer in patients with UC, particularly elderly patients.^21^, ^22^ The multivariate analysis in our study identified SR as an independent risk factor for recurrence in both groups, which can be associated with differences in pathological characteristics between patients who underwent SR and those who underwent TPC. However, our results also suggest that cancer recurrence had less influence on OS in elderly patients, even if they underwent SR. TPC for UC is safe regardless of age, but concomitant complications associated with other systemic diseases, such as heart or pulmonary disease, are prognostic factors in the surgical treatment of elderly patients, since they may increase surgical morbidity and mortality.^23^ Therefore, surgical procedures for elderly patients with UAC, including SR as an alternative option, should be carefully determined depending on performance status, extension of colitis, organ function, activities of daily living, and cancer progression.

This study has several limitations. First, this was a retrospective observational study that included data from patients who underwent treatment between 1980 and 2020. Consequently, the results may have been affected by changes in medical and surgical approaches over that time. While the potential influence of these factors on prognosis may not have been eliminated, we believe that our results reflect the reality of clinical practice. Second, this study had some missing data due to the retrospective nature of the data collection. A total of 136 patients were excluded due to missing data.

In conclusion, our study demonstrated that pathological factors such as residual tumor, nodal involvement and lymphovascular invasion had less impact on the risk of cancer recurrence in patients in the E‐UAC patients compared with the NE‐UAC patients. Patients in the E‐UAC group who underwent SR had worse RFS than those who underwent TPC, but there were no significant differences in DSS and OS. This suggests that SR may not invariably result in an unfavorable prognosis for elderly patients with UAC.

## AUTHOR CONTRIBUTIONS

K.N. is the corresponding author and contributed to the conception of the work, the analysis, the interpretation of data, writing the original draft, review, and editing. Y.M. and M.N. contributed to the analysis and interpretation of the data for the work and revised the work critically for important intellectual content. K.F., K.O., and U.N. contributed to the design of the work, data curation, and investigation. T.N. and S.I. contributed to data curation and investigation, the design of the work, and the interpretation of data, and revised the work critically for important intellectual content. Y.A. and K.S. contributed to the design of the work, data curation, and investigation. All authors approved the final version of the article to be published.

## FUNDING INFORMATION

This study was funded by the Japan Society of Laparoscopic Colorectal Surgery.

## CONFLICT OF INTEREST STATEMENT

The authors declare no conflicts of interest.

## ETHICS STATEMENT

Approval of the research protocol by an Institutional Reviewer Board: The study was approved by the Tokyo University Ethics Review Board (2019220NI‐[2]) and conformed to the ethical guidelines of the Japanese Government and the Declaration of Helsinki.

Informed Consent: The requirement for informed consent was waived due to the retrospective design of the study.

Registry and the Registration No. of the study/trial: N/A.

Animal Studies: N/A.

## Supporting information


**Table S1.** Recurrence patterns in 950 patients with stage 0–III UC‐associated colorectal cancer.
**Table S2.** Pathological risk factors for recurrence according to surgical procedure.
