# Supplementary material for: Prognostic value of surgical treatment in elderly patients with ulcerative colitis‐associated colorectal cancer: A subanalysis of a nationwide Japanese multicenter study
Source: Ann Gastroenterol Surg. 2024 Nov 12;9(3):486–95. doi: 10.1002/ags3.12885 (PMC12080187; doi:10.1002/ags3.12885)
Supplement: Supplementary file 1 — Table S1. Recurrence patterns in 950 patients with stage 0–III UC‐associated colorectal cancer. Table S2. Pathological risk factors for recurrence according to surgical procedure. [file AGS3-9-486-s001.docx]

Supplemental data

Table1. Recurrence patterns in 950 patients with stage 0–III UC-associated colorectal cancer

|  |  | E-UAC group | NE-UAC group | P-value |
| --- | --- | --- | --- | --- |
|  |  | N = 212 | N = 738 |  |
| Recurrence, n (%) |  | 19 (9.0%) | 100 (13.6%) | 0.07 |
|  |  |  |  |  |
| pStage, n (%)/N | 0 | 2 (2.9%)/69 | 3 (1.4%)/222 | 0.42 |
|  | I | 3 (4.6%)/65 | 17 (7.6%)/222 | 0.38 |
|  | II | 3 (7.5%)/40 | 21 (14.4%)/146 | 0.22 |
|  | III | 11 (29.0%)/38 | 59 (39.9%)/148 | 0.21 |
|  |  |  |  |  |
| Recurrence pattern, n (%) | Local | 3 (1.4%) | 25 (3.4%) | 0.11 |
|  | Lymph node | 3 (1.4%) | 15 (2.0%) | 0.59 |
|  | Liver | 1 (0.5%) | 21 (2.9%) | 0.02* |
|  | Lung | 7 (3.3%) | 17 (2.3%) | 0.43 |
|  | Peritoneum | 5 (2.4%) | 28 (3.8%) | 0.29 |

p, pathological; UC, ulcerative colitis

*Statistically significant (*P* < 0.05)

Table2. Pathological risk factors for recurrence according to surgical procedure

|  |  |  | E-UAC (n=212) |  |  |  | NE-UAC (n=738) |  |  |
| --- | --- | --- | --- | --- | --- | --- | --- | --- | --- |
|  |  |  | TPC | SR | P-value |  | TPC | SR | P-value |
|  |  |  | n=150 | n=62 |  |  | n=687 | n=111 |  |
| Recurrence | Present |  | 10 (6.7%) | 9 (14.5%) | 0.08 |  | 76 (11.7%) | 24 (26.7%) | <0.01* |
| Recurrent pattern | Local |  | 2 (1.3%) | 1 (1.6%) | 0.88 |  | 22 (3.4%) | 3 (3.3%) | 0.98 |
|  | Distant |  | 4 (2.6%) | 4 (6.5%) | 0.21 |  | 32 (4.9%) | 2 (2.2%) | 0.21 |
|  | Lymph node |  | 2 (1.3%) | 1 (1.6%) | 0.88 |  | 11 (1.7%) | 4 (4.4%) | 0.12 |
|  | Peritoneal |  | 3(2.0%) | 2 (3.2%) | 0.60 |  | 19 (2.9%) | 9 (10.0%) | <0.01* |
|  |  |  |  |  |  |  |  |  |  |
| Pathological risk factor for recurrence | Undifferentiated |  | 11 (7.3%) | 2 (3.2%) | 0.23 |  | 109 (15.9%) | 16 (14.4%) | 0.83 |
|  | pT(3-4) |  | 42 (28.0%) | 26 (41.9%) | 0.05* |  | 207 (30.1%) | 47 (42.3%) | <0.01* |
|  | pN(2-3) |  | 4 (2.6%) | 1 (1.65%) | 0.61 |  | 49 (7.1%) | 17 (15.3%) | <0.01* |
|  | Lymphatic invasion |  | 34 (22.6%) | 21 (33.9%) | 0.09 |  | 211 (30.7%) | 40 (36.0%) | 0.02* |
|  | Venous invasion |  | 31 (20.7%) | 18 (29.0%) | 0.18 |  | 177 (28.8%) | 39 (35.1%) | <0.01* |
|  | RM+ |  | 0 (0.0%) | 1 (1.6%) | 0.29 |  | 10 (1.5%) | 2 (2.2%) | 0.65 |

TPC; total proctocolectomy, SR; segmental resection, RM; resection margin
